# Ultrasound-assisted Maillard reaction of *Corynebacterium glutamicum* protein: Impact on structure, taste, and plant-based meat applications

**DOI:** 10.1016/j.ultsonch.2025.107424

**Published:** 2025-06-07

**Authors:** Jong Hyeon Han, Dong Hyun Keum, Hyun Ju Lee, Yea-Ji Kim, Hyun Su Jung, Do Hyun Kim, Hyuk Cheol Kwon, Dong-Min Shin, Sung Gu Han

**Affiliations:** a Department of Food Science and Biotechnology of Animal Resources, Konkuk University, Seoul 05029, South Korea; b Department of Food Science and Technology, Keimyung University, Daegu 42601, South Korea

**Keywords:** Corynebacterium glutamicum, Maillard reaction, Plant-based meat analogue, Taste, Ultrasound treatment, Umami

## Abstract

The Maillard reaction enhances sensory properties of foods by generating Maillard reaction products (MRPs). A major challenge for consumer acceptance of plant-based meat analogues is their lack of taste. *Corynebacterium glutamicum* produces various amino acids and proteins; however, its taste attributes were insufficient to substantially enhance the sensory characteristics of plant-based meat analogues. This study aimed to evaluate whether ultrasound-assisted Maillard reactions can improve the taste attributes of *C. glutamicum*-derived protein (CP). Additionally, the MRPs were applied in plant-based patties (PPs). Optimal Maillard reaction conditions were determined with a CP-saccharide mass ratio of 4:1, ultrasound power of 400 W, reaction time of 45 min, and a temperature of 70 °C. Structure, chemical changes, and taste attributes were assessed in the following samples: (1) CP (control), (2) CP-xylose (CPX), (3) CP-maltodextrin (CPM), (4) MRP of CP-xylose (MCPX), and (5) MRP of CP-maltodextrin (MCPM). The Maillard reaction significantly increased the grafting degree and browning intensity in MCPX and MCPM. Stable structural changes in MRPs were observed in MCPX and MCPM due to increased hydrogen bonds, hydrophobic interactions, and β-sheet content. MCPX and MCPM exhibited improved surface hydrophobicity, protein solubility, and thermal stability compared with other samples. The MRPs displayed reduced sourness and bitterness while enhancing umami taste. Incorporating MCPX and MCPM into PPs resulted in improved taste attributes and texture properties. Overall, this study demonstrated that ultrasound-assisted Maillard reaction induced structural and chemical changes and improved the taste attributes of CP, making it a suitable ingredient for plant-based meat analogues.

## Graphical abstract

**Figure:**
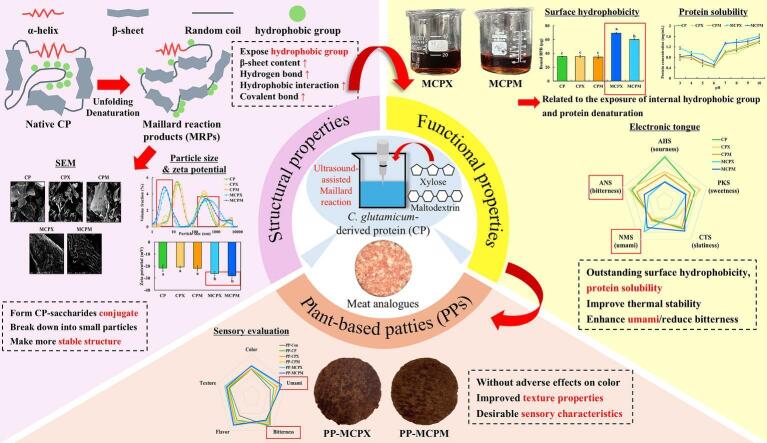

## 1 Introduction

Plant-based meat analogues (PMAs) are alternatives to traditional meat, formulated using plant proteins, water, oil, and polysaccharides. Many studies are currently being conducted to develop PMAs that replicate the taste, flavor, and texture of real meat while masking the beany flavor. Keum et al. [1] enhanced the liquid-holding capacity and juiciness of plant-based patties (PPs) by using a Pickering emulsion composed of different polysaccharides and pea protein. Zhang et al. [2] reported that the incorporation of mycelium derived from oyster mushrooms into PPs created a meat-like aroma while simultaneously reducing bitterness. Despite progress in replicating meat texture, the flavor and taste of PMAs remain significant obstacles to broader consumer adoption. To overcome these challenges, research must focus on creating authentic meat-like flavor and taste experiences while concurrently masking off-flavors and bitterness.

The Maillard reaction is a non-enzymatic chemical process whereby proteins, peptides, or amino acids are glycated with reducing sugars, resulting in the formation of Maillard reaction products (MRPs). The Maillard reaction is commonly divided into three main stages. In the initial stage, protein denaturation leads to the release of amino acids previously bound within the protein structure, thereby generating free amino acids. These free amino acids then interact with reducing sugars, undergo condensation, and transform into Amadori products [3]. In the following stage, the sugar is degraded and free amino acids are released from the Amadori products, leading to the formation of Strecker aldehydes [4]. Amadori products ultimately undergo polymerization, cyclization, dehydration, and decomposition. MRPs contain various compounds including furans and pyrazines, which are associated with meat flavor, as well as peptides that contribute to the umami taste [5,6]. Lysine and glutamate are key amino acids that contribute to flavor and taste development through the Maillard reaction. The ε-amino group of lysine is highly reactive in the Maillard reaction, leading to the formation of compounds such as pyrazines [7]. Free glutamate can impart umami taste and exhibits a synergistic effect in enhancing umami when combined with nucleotide-based flavor compounds [8]. In addition, glutamate can participate in the Maillard reaction, forming pyrazine derivatives such as 2-methyl-3,5-diethyl pyrazine that add meaty and roasted aromas, thereby enhancing the overall flavor profile alongside umami [9]. However, a single-step heat treatment induced minimal Maillard reaction between proteins and sugars due to the compact protein structures that shield reactive amino groups [10]. Therefore, both high temperatures and extended reaction times (often several days) are required in this single-step heat process for effective grafting [10].

Ultrasound, microwave, radiation, and high-pressure homogenization are well-established techniques for enhancing the Maillard reaction when combined with heat treatment [11]. Ultrasound treatment involves the formation, expansion, and collapse of small bubbles within the fluid, generating waves that raise internal pressure [12]. This process is known as the cavitation effect and deforms the protein structure, enhancing the Maillard reaction. In addition, ultrasound treatment significantly enhanced the formation of dicarbonyl compounds, key precursors of final MRPs, leading to increased levels of compounds such as organic acids and pyrazines [10]. The high-pressure conditions generated by ultrasound also promoted aldol-type condensation, resulting in unique volatile MRPs observed only under ultrasound-assisted Maillard reactions [10]. A previous study reported that MRPs generated from the ultrasound-assisted Maillard reaction of the chicken liver protein and xylose produced umami-related free amino acids, furan, and furfural resulting in enhanced umami taste and meat-like flavor [13]. In addition, MRPs derived from the Maillard reaction between myofibrillar protein and maltodextrin exhibited increased solubility, emulsifying properties, and thermal stability [14].

Extensive research on MRPs and their effects on taste highlighted their potential applications in various food products, including meat alternatives. Studies employed various proteins and peptides as reactants in the Maillard reaction. MRPs derived from soy protein hydrolysate and sucrase exhibited reduced bitterness by lowering the hydrophobic free amino acid content, while the formation of pyrazine compounds enhanced the flavor [15]. The Maillard reaction between oyster protein hydrolysate and *xylo*-oligosaccharides resulted in an increase in the content of sulfide-containing compounds, which contributed to a meat-like aroma [16]. Beyond animal and plant proteins, fungal and microalgae proteins, a type of microbial protein, have also been investigated in research on MRPs [17,18]. MRPs derived from *Lentinula edodes* protein and xylose resulted in a 69.29 % increase in saltiness enhancement and a 61.67 % increase in umami enhancement [17]. Microbial proteins, categorized into bacterial, microalgal, and fungal proteins, are increasingly recognized as functional additives in meat alternatives [19]. However, the potential of the Maillard reaction to enhance the taste and flavor of bacterial proteins remains unexplored. *Corynebacterium glutamicum* is a Gram-positive bacterium, with a high guanine–cytosine content and is generally regarded as safe [20]. Since 1957, research has been conducted on *C. glutamicum* to produce various amino acids, leading to its widespread utilization in industrial applications [20]. Indeed, *C. glutamicum* is well-suited for the synthesis of glutamate and lysine, producing approximately 700,000 tons of glutamate as an umami taste compound and 300,000 tons of lysine as a food additive [21]. In addition, *C. glutamicum* is suitable for protein production, particularly due to its low protease activity level in culture and potent ability to release proteins [22]. Thus, the food industry employs *C. glutamicum* to produce monosodium glutamate, a common food additive [23]. However, our preliminary analysis of PMAs containing *C. glutamicum*-derived protein (CP) showed no significant enhancement in flavor or umami after heating process. These data suggested that additional processing may be necessary to facilitate the release of free amino acids and the synthesis of flavor compounds from CP.

The potential of the Maillard reaction to enhance the specific characteristics of CP remains unexplored. Despite the growing interest in MRPs and bacterial proteins as quality enhancers, there is a lack of research assessing plant-based meat products that incorporate CP and its associated MRPs. Therefore, our objective was to enhance the flavor of CP through the Maillard reaction, capitalizing on its high glutamate and lysine content to improve the overall quality of plant-based meat alternatives (PMAs). In our study, ultrasound treatment was used to assist the Maillard reaction to improve efficiency and generate a greater variety of flavor compounds. We identified optimal ultrasound-assisted Maillard reaction conditions for CP in combination with xylose or maltodextrin. Subsequently, the sensory and physicochemical properties of the resulting MRPs were assessed for their suitability in PMA applications.

## 2 Materials and Methods

### 2.1 Materials

CP was provided by Professor Jung Kul Lee of Konkuk University. Following *C. glutamicum* cultivation, CP was purified using ethanol precipitation [24]. The fermentation broth was centrifuged (8,000 × g, 30 min), and ethanol (4 mL) was added to the supernatant, followed by overnight incubation at 4 °C. The mixture was then ultrasonically disrupted on ice (30 W, 1 sec intervals, 20 min). After a second centrifugation (8,000 × g, 30 min) to remove the supernatant, the precipitate was dissolved in water and freeze-dried to yield purified CP. Xylose and maltodextrin were purchased from Junsei Chemical Co. (Tokyo, Japan) and Kwangmung Food Co. (Seoul, Korea), respectively. Phthalaldehyde (OPA) and sodium tetraborate were purchased from Sigma-Aldrich Co. (St. Louis, MO, USA). β-mercaptoethanol and sodium dodecyl sulfate (SDS) were purchased from VWR Amresco (Randor, PA, USA). Sodium chloride and urea were purchased from Daejung (Busan, Korea) and Duksan General Science (Seoul, Korea), respectively. The bicinchoninic acid (BCA) assay kit and bromophenol blue were purchased from Thermo Fisher Scientific (Waltham, MA, USA). Textured pea protein (TPP) was purchased from Sotexpro Co. (Paris, France). κ-Carrageenan, salt, and beet powder were purchased from ESfood Co. (Gyeonggi, Korea). Potato starch was purchased from Daehan Flour Mills Co. (Seoul, Korea). Isolated pea protein was purchased from Hyangrim Co. (Seoul, Korea), whereas methyl cellulose was purchased from LOTTE Fine Chemical Co. (Ulsan, Korea). Canola and coconut oils were purchased from CJ Cheiljedang (Seoul, Korea) and Palmtop Vegeoil Products Sdn. Bhd. (Johor, Malaysia), respectively.

### 2.2 Preparation of Maillard reaction products and optimization of reaction conditions

The wet-heating method was employed to create MRPs synthesized from CP and saccharides using the ultrasonic-assisted Maillard reaction. The initial reaction conditions were based on those of a previous study [12]: the concentration of CP was 2 % (w/w); the ultrasound power was 400 W; the reaction time was 30 min; the treatment temperature was 70 °C. The CP-saccharide mixtures in a 50 mL conical tube were subjected to ultrasonic processing using a sonicator (Q700; Qsonica, CT, USA). The sonication parameters included a horn transducer with a diameter of 6 mm, an ultrasound output power range of 10–700 W, and an ultrasonic frequency of 20–25 kHz. To maintain a relatively constant temperature, a water bath was placed under the conical tube. The samples were preheated in the water bath (B2000-4-E; Benchmark Scientific, NJ, USA) for 5 min. Different CP-saccharide mass ratios (4:1, 2:1, 1:1, 1:2, and 1:4) were tested to identify the compositions that resulted in high grafting degree (GD) and browning intensity of MRPs.

Once the optimal composition of the CP-saccharide mass ratio was determined, the ultrasound power (0 W, 200 W, 400 W, and 600 W, with power density of 0 W/cm^3^, 9.29 W/cm^3^, 18.57 W/cm^3^, and 27.86 W/cm^3^, respectively), reaction time (15 min, 30 min, 45 min, and 60 min), and treatment temperature (50 °C, 60 °C, 70 °C, and 80 °C) were varied separately to assess the impacts of preparation conditions on the GD and browning intensity. The samples were named as follows: CP (control); CP-xylose mixture (CPX); CP-maltodextrin mixture (CPM); MRP of CP-xylose (MCPX); MRP of CP-maltodextrin (MCPM). All samples were freeze-dried for 72 h.

### 2.3 Characterization of MRPs

#### 2.3.1 Grafting degree and browning intensity

The GD of MRPs was determined using the OPA method as described previously Zhao et al. [12]. Eighty milligrams of OPA were dissolved in 2 mL of methanol, followed by mixing with 50 mL of 0.1 M sodium tetraborate buffer (pH 9.7), 200 μL of β-mercaptoethanol, and 5 mL of 20 % (w/w) SDS solution. The mixture was then diluted to a final volume of 100 mL with distilled water. These solutions were prepared immediately before use. Then, 100 μL of the sample solution (10 mg/mL) was added to 4 mL of OPA solution, and the resulting mixture was incubated at 35 °C for 2 min. The absorbance was measured at 340 nm using a UV–Vis spectrophotometer (BioTek Instruments, VT, USA). Each sample was measured three times. Native CP was used to prepare the control, and distilled water was used as the blank. The GD was calculated according to Eq. (1):

(1) $$\mathrm{GD}\;\left(\%\right)\;=\;(\left(C_{0}-C_{c}\right)/C_{0})\;\times \;100$$

C_0_: concentrations of free amino groups in native CP.

C_c_: concentrations of free amino groups in native CP-carbohydrate MRPs.

The browning intensity of the MRPs was evaluated using a previously described method [12]. The absorbance of samples (1 mg/mL) was measured at 294 nm and 420 nm using a UV–Vis spectrophotometer (BioTek Instruments, VT, USA). Each sample was measured three times.

#### 2.3.2 Fluorescence spectra

The samples were diluted with distilled water to a concentration of 1 mg/mL to detect the fluorescence intensity. The determination of fluorescence intensity was performed according to a previous study with minor modifications [25]. The fluorescence intensity of the solution was measured using a fluorescence spectrophotometer (Varioskan LUX; Thermo Fisher Scientific, MA, USA). The excitation wavelength was set to 347 nm, and emission wavelengths ranged from 350–500 nm. Each sample was measured three times.

#### 2.3.3 Fourier transform-infrared (FT-IR) spectroscopy

The secondary structure of the CP, CPX, CPM, MCPX, and MCPM samples were analyzed using an FT-IR spectrophotometer (FT/IR-4700; JASCO, Tokyo, Japan). The band within the 1600–1700 cm^−1^ range was deconvoluted using the PeakFit 4.12 software (SeaSolve Software Inc., Framingham, USA). The secondary structure content of samples was quantified as the Gaussian area under the curve of each associated peak: β-sheet (1615–1640 cm^−1^, 1690–1700 cm^−1^), α-helix (1650–1665 cm^−1^), β-turn (1665–1690 cm^−1^), and random coil (1640–1650 cm^−1^) [26]. Each sample was measured three times.

#### 2.3.4 Intermolecular forces

The intermolecular forces of CP, CPX, CPM, MCPX, and MCPM were determined by evaluating the protein solubility in different types of solvents [27]. The samples were dissolved in five different types of solutions (S1: 0.05 M NaCl; S2: 0.6 M NaCl; S3: 0.6 M NaCl + 1.5 M urea; S4: 0.6 M NaCl + 8 M urea; S5: 0.6 M NaCl + 8 M urea + 0.5 M β-mercaptoethanol) at a concentration of 2 mg/mL. The samples were centrifuged at 8,000 × g for 15 min. Protein solubility in the supernatant was measured using the BCA assay. Protein solubility was calculated according to a standard curve using bovine serum albumin. Differences in protein solubility between S1 and S2, S2 and S3, S3 and S4, and S4 and S5 indicated the ionic bonds, hydrogen bonds, hydrophobic interaction, and disulfide bonds, respectively. Each sample was measured three times.

#### 2.3.5 Particle size and zeta-potential

The particle size and zeta potential of CP, CPX, CPM, MCPX, and MCPM samples were measured using dynamic light scattering (DLS) analysis. The samples were dissolved in distilled water at a concentration of 1 mg/mL and analyzed using a Zetasizer Nano ZS instrument (Malvern Instruments Ltd., Malvern, UK). The measurements were taken at an angle of 90°, a wavelength of 633 nm, and temperature of 25 °C. Each sample was measured three times.

#### 2.3.6 Scanning electron microscopy (SEM)

The freeze-dried samples were affixed to a conductive adhesive and coated with gold using an ion sputter coater (MCM-200, SEC Co., Ltd., Gyeonggi, Korea). The microstructure was examined at a magnification of 300 × using a scanning electron microscope (TM3000, Hitachi Ltd., Tokyo, Japan).

### 2.4 Evaluation of MRPs

#### 2.4.1 Surface hydrophobicity

The surface hydrophobicity was assessed by adding 200 μL of bromophenol blue (BPB, 1 mg/mL) solution to 1 mL of the sample (1 mg/mL). The mixture was incubated for 10 min and then centrifuged at 8,000 × g for 15 min. The supernatant was diluted ten-fold, and the absorbance was measured at 595 nm using a UV–Vis spectrophotometer (BioTek Instruments, VT, USA). Samples were measured three times. Distilled water was used as a blank, and surface hydrophobicity was calculated according to Eq. (2):

(2) $$\mathrm{Bound}\;BPB:\;\left(200\;\mu g\;\times \;\left({\mathrm{Ab}}_{\mathrm{blank}}-{\mathrm{Ab}}_{\mathrm{sample}}\right)\right)/{\mathrm{Ab}}_{\mathrm{blank}}$$

#### 2.4.2 pH-dependence of protein solubility

The protein solubility was measured using the method described by Zhao et al. [12] with slight modifications. The samples were diluted with distilled water to a concentration of 2 mg/mL. The pH of the MRP solution was then adjusted to a range of 3 to 10 by adding NaOH or HCl solutions. The adjusted samples were centrifuged at 8,000 × g for 15 min. The BCA assay was used to quantify the protein content in the supernatant. Protein solubility was calculated according to the standard curve using bovine serum albumin. Each sample was measured three times.

#### 2.4.3 Differential scanning calorimeter (DSC)

The thermal properties of CP, CPX, CPM, MCPX, and MCPM samples were evaluated using a differential scanning calorimeter (DSC 4000; PerkinElmer, MA, USA). The samples were diluted with distilled water to a concentration of 10 mg/mL and placed in an aluminum pan. A sealed, empty pan was used as the reference. The temperature was raised from 20 °C to 80 °C at a rate of 10 °C/min. Enthalpy change (delta H), onset, peak, and end temperatures were calculated using the data analysis software Pyris (PerkinElmer, MA, USA). Each Sample was measured three times.

#### 2.4.4 Electronic tongue (E-tongue)

The taste attributes of CP, CPX, CPM, MCPX, and MCPM were measured using an E-tongue (Astree II; Alpha MOS, Occitanie, France) equipped with five taste sensors (AHS for sourness, PKS for sweetness, CTS for saltiness, NMS for umami, and ANS for bitterness) and two standard sensors (SCS and CPS). The sensors were conditioned and calibrated with 0.01 M hydrochloric acid solution. The samples were diluted to a concentration of 10 mg/mL in distilled water. Subsequently, 25 mL of each solution was placed in the autosampler of the E-tongue. Each sample was measured five times for 120 s at 25 °C. To prevent cross-contamination between samples during analysis, the sensors were washed two times with distilled water for 10 s each time after testing. The five taste sensors analyzed the overall taste profile of the samples and quantified their responses into relative taste scores ranging from 0 to 10, using the AlphaSoft 17 software (Alpha MOS, Occitanie, France).

### 2.5 Preparation of PPs

To evaluate the role of CP, CPX, CPM, MCPX, and MCPM in PPs, six formulations of PP were prepared (Table S1): PP supplemented with CP 1 % (w/w) (PP-CP, control); PP supplemented with CPX 1 % (w/w) (PP-CPX); PP supplemented with CPM 1 % (w/w) (PP-CPM); PP supplemented with MCPX 1 % (w/w) (PP-MCPX); PP supplemented with MCPM 1 % (w/w) (PP-MCPM). A control group (PP-Con) was manufactured without CP and MRPs. TPP was hydrated for 1 h at 4 °C using distilled water. TPP was then mixed with the ingredients for 6 min. Subsequently, 90 g of the mixture was used to produce patties using a patty presser (manual burger press 4″, Spikomat Ltd., Nottingham, UK). To evaluate the physicochemical properties of cooked patties, samples were cooked using an electric pan (DW-1530, Daewon Home Electric Co., Ltd., Gyeonggi, Korea) at 150 °C for 3 min per side until the internal temperature of patties reached 80 °C. The cooked patties were allowed to equilibrate at room temperature for 30 min before the measurement of the surface color and texture properties.

### 2.6 Physicochemical and sensory characteristics of PPs

#### 2.6.1 Color, rheological properties, and texture profile analysis of PPs

The color of raw and cooked patties was measured using a colorimeter (CR-210; Konica Minolta, Ltd., Osaka, Japan). The colorimeter was calibrated using a white plate (L* = +97.27, a* = +5.21, b* = − 3.40). The color was then presented as L* (lightness), a* (redness), and b* (yellowness). Each sample was measured six times.

The rheology properties of samples were assessed using a rheometer (MCR 92, Anton Paar, Graz, Austria) equipped with a 25 mm-diameter parallel plate. Raw patties were loaded onto a static plate with a 1 mm gap between the plates. Apparent viscosity was measured at 25 °C with shear rates from 0.1 to 100 s^−1^. The linear viscoelasticity region was determined by conducting strain sweep tests with strain ranging from 0.01 % to 10 % at a frequency of 1 Hz. The frequency sweep test was performed under the following conditions: temperature, 25 °C; frequency, 0.1–100 rad/s; strain, 1.0 %. During each test, the storage modulus (G′) as well as loss modulus (G″) were measured. Each sample was measured in triplicate.

The texture profile analysis (TPA) of PPs was conducted using a texture analyzer (TA-XT Plus, Stable Micro Systems Ltd., Surrey, Goldaming, UK) equipped with a 40 mm cylindrical probe (P/40). Cooked patties were sectioned into uniform pieces measuring 1.5 cm × 1.5 cm × 1 cm. Texture parameters including hardness, springiness, cohesiveness, chewiness, and gumminess were evaluated. Measurements of the sample were performed under the following conditions: pre-test speed 2.0 mm/s; test speed 1.0 mm/s; post-test speed 1.0 mm/s; force 5 g. Five measurements were taken for each PP sample.

#### 2.6.2 Sensory evaluation of PPs

Sensory evaluation of PPs was conducted according to our previously described method [26]. Seven trained panelists (four males and three females, aged 24–34 years) participated in the evaluation. In the initial training sessions, we introduced the patties to the panelists to help them become familiar with specific sensory attributes to be assessed including umami, bitterness, flavor, texture, and color. Panelists underwent three training sessions using PP samples, each session lasting 20–30 min, over a two-week period prior to the formal evaluation. For the sensory evaluation, each sample was cut into 1.5 cm × 1.5 cm × 1 cm pieces, and a 3-digit random number was assigned to each sample. After evaluating each sample, the panelists cleaned their palates with water. Intensities for umami, bitterness, flavor, texture, and color were assessed using a 9-point rating scale (1 = not at all, 9 = extremely strong). The Institutional Review Board approved the procedure for sensory evaluation (KKUIRB-202411-HR-019). All participants provided informed written consent at the beginning of the study for their data to be used and analyzed.

### 2.7 Statistical analysis

All data are expressed as the mean ± standard deviation. Statistical analyses were performed using the SPSS-PASW statistics software (version 22.0; SPSS Inc., IL, USA). Statistical significance was analyzed using one-way analysis of variance (ANOVA). Duncan’s multiple range post-hoc test was used to identify significant differences (*P* < 0.05) between groups.

## 3 Results and Discussion

### 3.1 Optimization of ultrasound-assisted Maillard reaction conditions

**Fig. 1:**
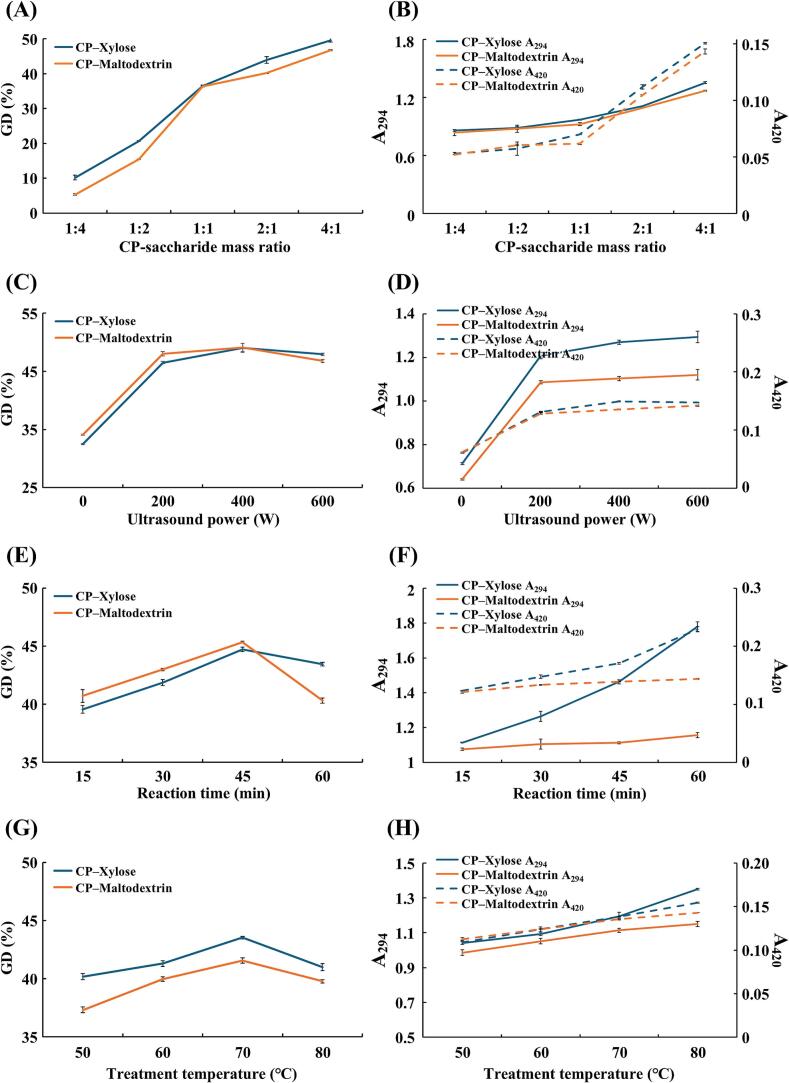
Influence of different ultrasound treatment parameters on the grafting degree and browning intensity of *C. glutamicum*-derived protein (CP) in the Maillard reaction. (A–B) CP–saccharide mass ratio (Fixed CP concentration: 2 wt%; ultrasound power: 400 W; reaction time: 30 min; treatment temperature: 70 ℃), (C–D) ultrasound power (CP concentration: 2 wt%; saccharide concentration: 0.5 wt%; reaction time: 30 min; treatment temperature: 70 ℃), (E–F) reaction time (CP concentration: 2 wt%; saccharide concentration: 0.5 wt%; ultrasound power: 400 W; treatment temperature: 70 ℃), and (G–H) treatment temperature (CP concentration: 2 wt%; saccharide concentration: 0.5 wt%; ultrasound power: 400 W; reaction time: 30 min). The error bars indicate standard deviations (n = 3). Each colored line indicates a different sample.

Protein grafting during the Maillard reaction results in pigment formation and browning, both serving as indicators of this complex process. To assess the degree of grafting, the OPA method was used. Browning intensity can be assessed by measuring absorbance at 294 nm (A_294_) for early MRPs and at 420 nm (A_420_) for final MRPs [28]. Our study aimed to optimize ultrasound parameters and conditions for the Maillard reaction in our samples by measuring GD and browning intensity. The results showed that the GD of MRPs tended to increase with higher concentrations of CP in the CP–saccharide mass ratio, reaching the highest value at a ratio of 2 % CP and 0.5 % saccharide (i.e., xylose or maltodextrin) (Fig. 1A). In contrast, as the content of saccharides increased, the GD value decreased. This decrease likely occurred because high saccharide content increased the fluid viscosity, which inhibited the Maillard reaction by restricting the movement and binding of internal molecules [29]. Both A_294_ and A_420_ of MRPs exhibited a similar trend to GD (Fig. 1B). Thus, the optimal mass ratio of 2 % CP and 0.5 % saccharide was selected for further evaluation.

During the Maillard reaction, ultrasound transmitted additional energy through the cavitation effect, inducing protein unfolding and enhancing the release of free amino acids from the protein structure. This process, along with the heating process, could enhance grafting. In addition, ultrasound treatment induced intermolecular movement and shortened the distance between reactants by creating waves throughout the fluid [30]. The MRPs treated with ultrasound exhibited higher GD values than those under the 0 W condition (Fig. 1C), indicating that ultrasound treatment enhanced the Maillard reaction between CP and saccharides. The GD of MRPs increased with ultrasound power up to 400 W, but the GD value decreased at 600 W. Ultrasound power up to 400 W enhanced the Maillard reaction between CP and saccharides. However, excessively high intensity caused extensive aggregation of external hydrophobic groups exposed by the unfolding of proteins, resulting in the reduction of the number of free amino acids and inhibiting the Maillard reaction [12]. Consistent with our findings, a previous study reported that the GD value ​​of whey protein and gum acacia increased with ultrasound power up to 500 W but decreased at 600 W [11]. The MRPs treated with ultrasound exhibited significantly higher A_294_ and A_420_ values compared to the 0 W condition (Fig. 1D), suggesting that heat treatment alone was insufficient to effectively induce the Maillard reaction of CP. The A_294_ and A_420_ values increased with rising ultrasound power, reaching their highest values at 600 W (Fig. 1D). However, the difference in A_294_ and A_420_ values between 400 W and 600 W of ultrasound power was negligible. Therefore, considering both GD and browning intensity, 400 W of ultrasound power was selected as the optimal condition for the Maillard reaction.

Under various reaction time and temperature conditions, the highest GD of MRPs was observed at 45 min and 70 °C (Fig. 1E and 1G). Optimal reaction time and temperature were thought to enhance grafting by supplying additional energy and facilitating contact between the saccharides and the free amino groups. However, excessive reaction time (60 min) and treatment temperature (80 °C) might lead to protein aggregation, resulting in reduced accessibility of amino acids in the Maillard reaction (Fig. 1E and 1G). Meanwhile, A_294_ and A_420_ values tended to increase with prolonged reaction time and higher temperature, with xylose exhibiting a greater increase compared to maltodextrin (Fig. 1F and 1H). During the Maillard reaction, monosaccharides exhibited higher reactivity than polysaccharides, leading to more active browning and protein polymerization processes [31]. In this study, MRPs derived from xylose, a monosaccharide, had a more pronounced impact on browning intensity than those from maltodextrin, a polysaccharide. However, a high A_420_ value indicates the production of numerous advanced glycation end products, which can contribute to various diseases such as kidney dysfunction and cancer [32]. Therefore, considering both GD and browning intensity, the optimal reaction time and temperature were selected as 45 min and 70 °C, respectively.

Collectively, the optimal conditions for the Maillard reaction were selected as follows: CP–saccharide mass ratio of 4:1 (CP: 2 %, saccharide: 0.5 %); ultrasound power of 400 W; reaction time of 45 min; treatment temperature of 70 °C.

### 3.2 Characterization of MRPs structure

#### 3.2.1 Grafting degree analysis, browning intensity, and fluorescence spectra of MRPs

**Fig. 2:**
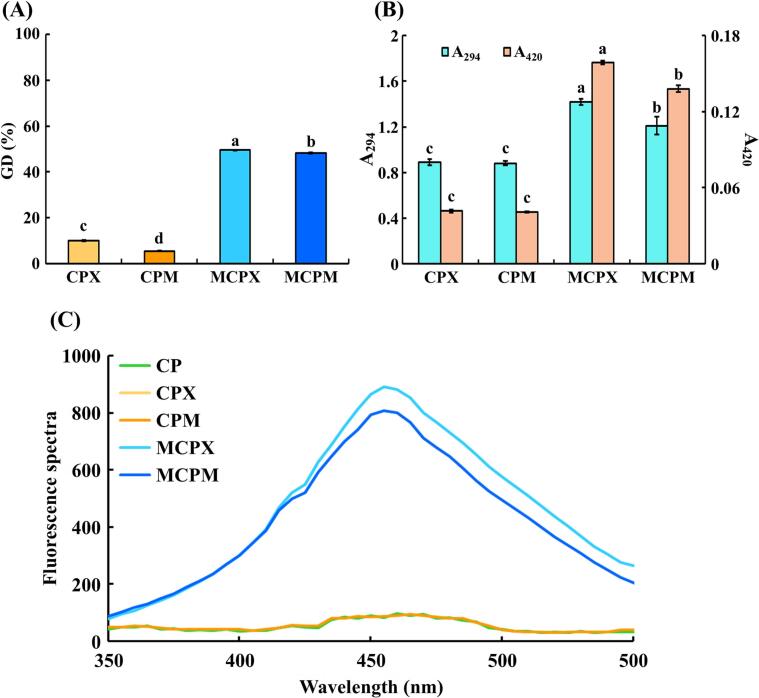
Degree of Maillard reaction between *C. glutamicum-*derived protein and saccharides. (A) Grafting degree, (B) browning intensity, and (C) fluorescence spectra. The error bars indicate standard deviations (n = 3). Different letters denote statistical differences (*P* < 0.05). Each colored line indicates a different sample. CP: *C. glutamicum*-derived protein, CPX: CP and xylose mixture, CPM: CP and maltodextrin mixture, MCPX: Maillard reaction product synthesized from CP and xylose, MCPM: Maillard reaction product synthesized from CP and maltodextrin.

Fluorescent compounds, early intermediate MRPs, could be used to detect the occurrence of the Maillard reaction [33]. Xylose and maltodextrin, monosaccharide and polysaccharide saccharide sources respectively, were selected for the Maillard reaction due to their common use in other studies [13,14]. Then, to evaluate whether CP undergoes a proper Maillard reaction with monosaccharides or polysaccharides under the selected conditions of ultrasound-assisted wet heating, GD, browning intensity, fluorescence spectra, and the visual appearance of MRPs were measured (Fig. 2 and Fig. S1). Through the Maillard reaction, MCPX and MCPM exhibited significantly higher GD values compared with CPX and CPM, respectively (*P* < 0.05) (Fig. 2A). The GD value of MCPX was slightly higher than that of MCPM. This might be due to xylose, being a monosaccharide, promoting protein polymerization processes and inducing a higher GD than that in maltodextrin [31]. Collectively, under the selected conditions, ultrasound treatment facilitated effective grafting during the Maillard reaction with both xylose and maltodextrin.

Variations in browning intensity and visual appearance resulting from the optimized Maillard reaction conditions were determined (Fig. 2B and Fig. S1). Absorbance at 294 nm was used to quantify the content of MRPs in the initial stage of the Maillard reaction, including Amadori products, Heyns products, and dicarbonyl compounds. These compounds act as precursors to various flavor compounds, enhancing the taste and aroma of foods. Absorbance at 420 nm measures the content of melanoidins generated in the later stages of the Maillard reaction. These brown and nitrogen-containing MRPs could influence the appearance of foods [34]. In our data, MCPX and MCPM exhibited significantly higher A_294_ and A_420_ values compared with CPX and CPM, respectively, with MCPX exhibiting the highest values (*P* < 0.05). Hence, the optimal Maillard reaction conditions were deemed appropriate, as evidenced by increased values of A_294_ and A_420_ for MCPX and MCPM, potentially imparting positive effects on food products.

Fluorescent substances emerged during the initial stage of the Maillard reaction. These substances acted as precursors to browning compounds and were detectable at an excitation wavelength of 347 nm, with emission occurring between 350–500 nm [35,36]. As shown in Fig. 2C, setting the excitation wavelength to 347 nm resulted in strong fluorescence at 450–460 nm due to the fluorescent compounds generated by the Maillard reaction. Among the samples, MCPX and MCPM exhibited higher fluorescence intensity than CP, CPX, and CPM, with MCPX demonstrating the highest fluorescence intensity (Fig. 2C). This trend aligned with the results of GD (Fig. 2A). Furthermore, following the reaction, the emission wavelength of the maximum fluorescence intensity (λ_max_) tended to decrease from 460 nm to 455 nm (i.e., blue shift). The increase in fluorescence intensity and blue shift were attributed to the accelerated Maillard reaction, leading to the generation of additional fluorescent substances such as pyridine, pentoside, argpyrimidine, and pyrrole [35]. In addition, variations in the internal fluorescence of MCPX and MCPM were influenced by changes in tryptophan, suggesting a more active induction of protein structural changes [37]. Therefore, under optimal Maillard reaction conditions, MCPX and MCPM produced fluorescent compounds and exhibited a blue shift, resulting from protein structure modification.

#### 3.2.2 Secondary structure and intermolecular forces of MRPs

**Fig. 3:**
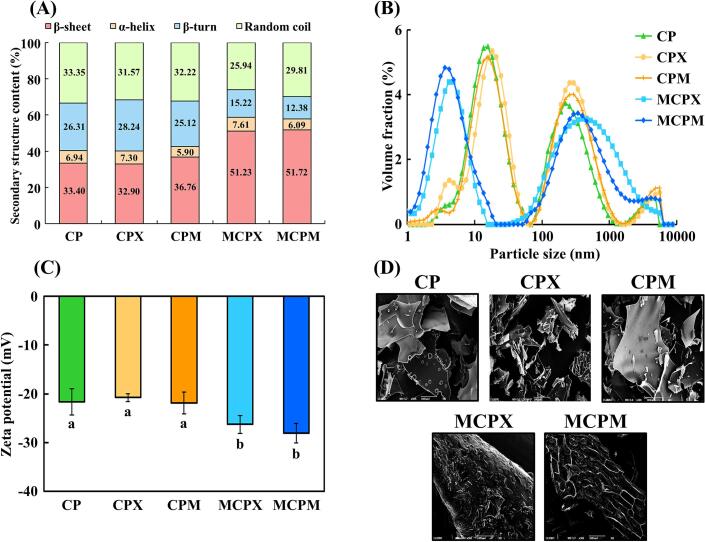
Structural characteristics of *C. glutamicum-*derived protein and Maillard reaction products (MRPs). (A) Secondary structure, (B) particle size distribution, (C) zeta potential, and (D) scanning electron microscopy images. Each colored line indicates a different sample. The error bars indicate standard deviations (n = 3). Different letters denote statistical differences (*P* < 0.05). CP: *C. glutamicum*-derived protein, CPX: CP and xylose mixture, CPM: CP and maltodextrin mixture, MCPX: MRPs synthesized from CP and xylose, MCPM: MRPs synthesized from CP and maltodextrin.

Fluorescence spectra indicating Maillard-induced CP structural changes led to an investigation using FT-IR spectra, protein secondary structure, and intermolecular force analyses (Fig. 3A, Table 1). With the combined ratios of the β-sheet, α-helix, and β-turn in all samples accounting for approximately 60 %, the protein structure was considered stable and orderly [38] (Fig. 3A). CP exhibited a composition of 33.40 % β-sheet, 6.94 % α-helix, 26.31 % β-turn, and 33.35 % random coil. Despite CPX and CPM undergoing only physical mixing without heating or ultrasound treatment, increase in the the α-helix and β-sheet contents were observed. In MCPX, the ratio of β-turn and random coil decreased to 15.22 % and 25.94 %, respectively, while the β-sheet content increased to 51.23 %. Likewise, in MCPM, the ratios of β-turn and random coil decreased to 12.38 % and 29.81 %, respectively, while the β-sheet content increased to 51.72 %. The observed changes could be due to heating, ultrasound treatment, and carbohydrate attachment, which increasd β-sheet content by inducing protein deformation, unfolding, and solubilization. This process exposed hydrophobic and sulfhydryl regions, thereby facilitating protein–saccharide conjugation [39]. Additionally, the increase in β-sheet content suggested that the unstable hydrogen bond structure of β-turn might be converted to the more stable β-sheet structure [30]. Collectively, these data suggest that the optimal Maillard reaction condition could induce denaturation in MCPX and MCPM by facilitating the transition from β-turn to β-sheet structures.

**Table 1:** Intermolecular forces of *C. glutamicum*-derived protein and Maillard reaction products (MRPs).

Chemical interactions		CP	CPX	CPM	MCPX	MCPM
Intermolecular forces (mg/mL)	Ionic bonds	0.05 ± 0.01^Dbc^	0.03 ± 0.01^Dc^	0.04 ± 0.01^Dbc^	0.05 ± 0.01^Dab^	0.07 ± 0.01^Da^
	Hydrogen bonds	0.23 ± 0.01^Cb^	0.24 ± 0.02^Cb^	0.24 ± 0.03^Cb^	0.58 ± 0.01^Ca^	0.59 ± 0.01^Ca^
	Hydrophobic interaction	0.33 ± 0.02^Bd^	0.32 ± 0.02^Bd^	0.44 ± 0.01^Bc^	0.69 ± 0.00^Bb^	0.76 ± 0.03^Aa^
	Disulfide bonds	1.49 ± 0.00^Aa^	1.48 ± 0.04^Aa^	1.34 ± 0.03^Ab^	0.75 ± 0.00^Ac^	0.65 ± 0.03^Bd^

CP: *C. glutamicum*-derived protein, CPX: CP and xylose mixture, CPM: CP and maltodextrin mixture, MCPX: MRPs synthesized from CP and xylose, MCPM: MRPs synthesized from CP and maltodextrin.

^A–D^ Means values in the same column are significantly different (*P* < 0.05).

^a–d^ Means values in the same row are significantly different (*P* < 0.05).

Data are presented as mean ± standard deviation (n = 3).

Intermolecular forces were measured to verify the chemical bond alterations between CP and xylose or maltodextrin during the Maillard reaction (Table 1). In CP, disulfide bonds were dominant, followed by hydrophobic interaction, hydrogen bonds, and ionic bonds. In comparison to CP, CPX did not show significant differences in any interactions, while CPM exhibited significantly more hydrophobic interactions and fewer disulfide bonds (*P* < 0.05). Consistent with previous findings [40], where short blending of maltodextrin and casein increased hydrophobic interactions, we observed that short mixing of CP and maltodextrin exposed hydrophobic groups, enhancing these interactions. MCPX and MCPM exhibited significant increases in hydrogen bonds and hydrophobic interaction, along with a significant decrease in disulfide bonds (*P* < 0.05). Hydrogen bonds played a crucial role in maintaining protein secondary structure, and an increase in hydrogen bonds indicated enhanced mutual bonding between CP, saccharides, and water [41]. The Maillard reaction causes protein deformation, unfolding, and solubilization, which exposes hydrophobic groups and promotes hydrophobic interactions through carbonyl-amino condensation. Additionally, the Maillard reaction could break disulfide bonds within polypeptide chains, releasing them to the exterior, where they might be converted into sulfhydryl groups [38]. These insights into intermolecular forces suggested potential mechanisms by which the Maillard reaction affected CP and saccharides (e.g., xylose or maltodextrin).

#### 3.2.3 Particle size, zeta-potential, and scanning electron microscopy of MRPs

Particle size, zeta potential, and surface morphology are key indicators for evaluating the stability of protein and protein solubility. In addition, variations in particle size could be used to evaluate structural characteristics such as the conjugation of CP and saccharides during the Maillard reaction [16]. The particle size of native CP exhibited two peaks at approximately 20 nm and 200 nm (Fig. 3B). In CPX and CPM, the positions of these peaks shifted slightly to the right, suggesting that some conjugation occurred through simple mixing of CP and saccharides. MCPX and MCPM showed a rightward shift of the peak at 200 nm compared with CPX and CPM, indicating that covalent bonds between CP and saccharides were formed due to heating and ultrasound treatment, resulting in larger structures [39]. Conversely, in MCPX and MCPM, the peak at 20 nm shifted to the left compared to that in CP, CPX, and CPM. Ultrasound treatment decomposed the structure of CP through shear stress, internal pressure, and dynamic agitation in the solution, reducing the degree of molecular aggregation. In other words, during the Maillard reaction, CP combined with saccharides to form large molecules while simultaneously breaking down CP into smaller particles [39,42]. Our findings suggest that CP underwent a Maillard reaction with xylose or maltodextrin, resulting in the formation of covalent conjugates and simultaneous production of smaller molecules.

To evaluate the structural stability of the MRP solutions based on surface potential, the zeta potential was measured (Fig. 3C). CPX and CPM exhibited no significant difference compared to CP (*P* > 0.05), indicating that simple mixing of CP with xylose or maltodextrin did not alter the surface charge. Meanwhile, the zeta potential values of MCPX and MCPM were significantly more negative than those of CP, CPX, and CPM (*P* < 0.05). These data suggested a reduction in positively charged amino acids due to the covalent bonding of negatively charged xylose or maltodextrin with CP [43]. Furthermore, the reduction in zeta potential might result from the exposure of internal negatively charged groups due to protein unfolding during the Maillard reaction. This exposure amplified electrostatic repulsion, which could benefit various applications [12]. Thus, during the Maillard reaction, CP bound with xylose or maltodextrin, increasing the negative charge on the surface and forming a more stable dispersed phase.

The surface morphology of the MRPs was verified using SEM (Fig. 3D). CP, CPX, and CPM exhibited rough, fragmented, and slightly porous structures. A porous structure was closely related to solubility, suggesting that CP itself had excellent solubility [39]. Meanwhile, MCPX formed a macromolecular irregular and highly porous structure that facilitated interaction with water, enhancing solubility [39]. MCPM formed a large structure through cross-linking between CP and saccharide, with numerous pores distributed throughout the structure. Collectively, the SEM results indicated that CP conjugated with saccharides during the Maillard reaction, resulting in structural changes through modifications in the functional groups, secondary structure, and particle size.

### 3.3 Evaluation of Maillard reaction products functionality

#### 3.3.1 Surface hydrophobicity of MRPs

**Fig. 4:**
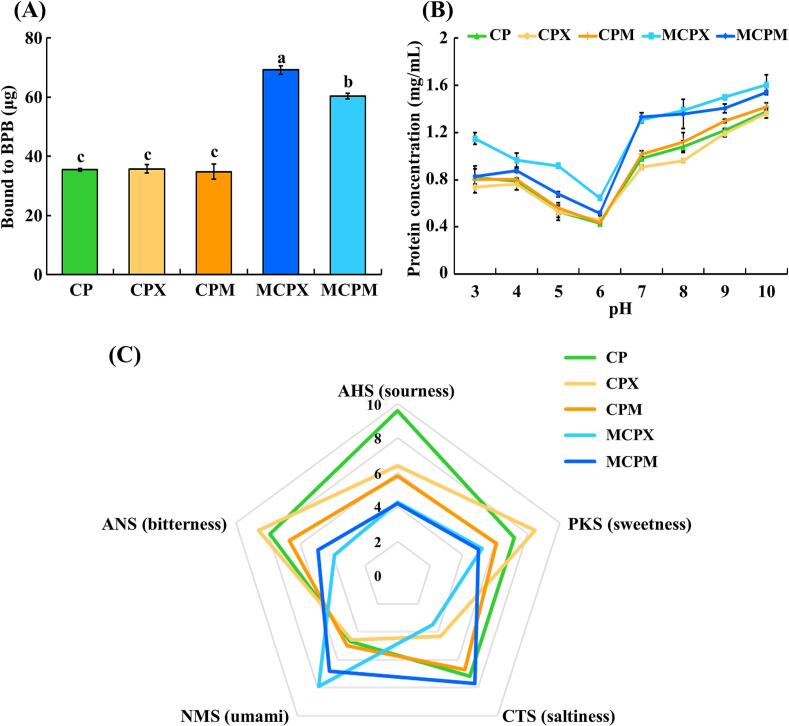
Protein functionality of *C. glutamicum-*derived protein and Maillard reaction products (MRPs). (A) Surface hydrophobicity, (B) protein solubility at pH 3–10, and (C) electronic tongue. The error bars indicate standard deviations (n = 3). Different letters denote statistical differences (*P* < 0.05). Each colored line indicates a different sample. CP: *C. glutamicum*-derived protein, CPX: CP and xylose mixture, CPM: CP and maltodextrin mixture, MCPX: MRPs synthesized from CP and xylose, MCPM: MRPs synthesized from CP and maltodextrin.

Surface hydrophobicity represents the presence of hydrophobic amino acid groups on the exterior of a protein. Hydrophobic residues on the exterior of proteins played a crucial role in modulating surface tension, foaming properties, and emulsifying properties [39]. Therefore, surface hydrophobicity was measured to verify the altered characteristics of CP resulting from the Maillard reaction. Surface hydrophobicity was quantified by measuring the absorbance resulting from the interaction between the surface hydrophobic groups and BPB [44]. The surface hydrophobicity of CPX and CPM showed no significant differences compared with that of CP (Fig. 4A). However, MCPX and MCPM exhibited significantly higher surface hydrophobicity than CP, CPX, and CPM (*P* < 0.05) (Fig. 4A). During heating, the hydrophobic groups of CP were exposed due to protein unfolding, while saccharides likely underwent modifications. Following protein unfolding, CP might be aggregated, driven by the crosslinking through hydrophobic interactions between the newly exposed hydrophobic groups and the modified saccharides [42]. In addition to heating, ultrasound treatment also promoted protein unfolding through cavitation effects, exposing internal hydrophobic groups of CP and thereby increasing the surface hydrophobicity of MCPX and MCPM. These observations were consistent with the results of FT-IR spectra and intermolecular forces, indicating increased hydrophobic interaction and β-sheet content in MCPX and MCPM (Fig. 3A, Table 1).

#### 3.3.2 pH-dependent protein solubility of MRPs

The water solubility of protein can influence the appearance of foods and enhance its gelling, foaming, and emulsification properties [12]. The protein solubilities of CP, CPX, CPM, MCPX, and MCPM at pH 3 to 10 were determined (Fig. 4B). The solubility of CP was the lowest at pH 6, indicating that the isoelectric point of CP was near this value. Other groups also exhibited the lowest solubility at pH 6, suggesting that the conjugation between CP and saccharides did not inhibit aggregation at the isoelectric point [12]. Following heating and ultrasound treatment, the solubility of MCPX and MCPM increased across all pH ranges. The increased solubility likely resulted from covalent bonding between CP and saccharides, which enhanced protein–water binding and suppressed aggregation under unfavorable temperature and net-charge conditions [11]. Meanwhile, according to the DLS results, the particle sizes of MCPX and MCPM were smaller than those of other samples (Fig. 3B). Ultrasound-induced high shear rates resulted in protein unfolding, which exposed hydrophobic groups and formed small-sized soluble protein aggregates from hydrophobic precipitates [45]. These soluble protein aggregates facilitated the interaction between the protein and water, thereby enhancing solubility. In addition, an increase in β-sheet content and surface hydrophobicity during the Maillard reaction correlated with this phenomenon (Fig. 3A, Fig. 4A). Collectively, the ultrasound-assisted Maillard reaction increased the solubility of MRPs by enhancing protein–water binding and forming small-sized soluble protein aggregates.

#### 3.3.3 Differential scanning calorimeter of MRPs

DSC can be used to assess the thermal stability of CP by monitoring heat flow variations with temperature changes. DSC measured the heat energy required for protein transitions, providing insights into the thermal properties of the protein sample [46]. The alterations in the thermal stability of CP resulting from the Maillard reaction were determined (Table 2). All samples exhibited peak temperatures within the range of 60–61 °C, indicating that thermal transitions occurred [12]. The peak temperatures of CPX and CPM did not significantly differ from that of CP (*P* > 0.05), suggesting that the presence of saccharides in the mixture did not affect the molecular environment of CP. MCPX and MCPM had significantly higher peak temperatures compared with CP, with MCPX exhibiting the highest peak temperature (*P* < 0.05). A high peak temperature, where a protein underwent denaturation due to heat, indicated excellent thermal stability [46]. Thermal stability was related to GD; consequently, MCPX and MCPM showed elevated peak temperatures due to high GD values through the Maillard reaction [12]. Among them, MCPX, which likely exhibited more desirable cross-linking and protein modification, was considered to have excellent thermal stability. The delta H values of MCPX and MCPM were significantly lower than those of CP, CPX, and CPM (*P* < 0.05). Delta H represents the enthalpy change associated with the transition of a protein. A low delta H indicated the formation of covalent bonds between the protein and saccharides during the conjugation reaction while simultaneously disrupting the intramolecular forces within the protein [47]. In this study, the decrease in delta H during the Maillard reaction was attributed to the formation of covalent bonds and the exposure of hydrophobic groups within CP. This result was associated with an increase in β-sheet content, and surface hydrophobicity (Fig. 3A, Fig. 4A). The decrease in delta H and increase in thermal stability was related to the characteristics of the protein, such as solubility, emulsion stability, and foaming stability [47]. Taken together, the thermal stability of MCPX and MCPM was enhanced through protein modification during the Maillard reaction, suggesting improved protein functions.

**Table 2:** Thermal stability of *C. glutamicum*-derived protein and Maillard reaction products (MRPs).

	CP	CPX	CPM	MCPX	MCPM
Onset temperature (℃)	57.80 ± 0.13	57.77 ± 0.02	57.79 ± 0.12	57.92 ± 0.04	57.85 ± 0.10
Peak temperature (°C)	60.25 ± 0.17^c^	60.59 ± 0.49^bc^	60.43 ± 0.26^c^	61.33 ± 0.53^a^	61.20 ± 0.23^ab^
End temperature (℃)	69.30 ± 0.51^a^	69.59 ± 0.53^a^	69.32 ± 0.34^a^	68.09 ± 0.30^b^	68.39 ± 0.16^b^
Delta H (mJ/g)	46.60 ± 4.35^a^	48.43 ± 9.42^a^	47.0 ± 3.92^a^	29.27 ± 5.10^b^	33.20 ± 0.87^b^

CP: *C. glutamicum*-derived protein, CPX: CP and xylose mixture, CPM: CP and maltodextrin mixture, MCPX: MRPs synthesized from CP and xylose, MCPM: MRPs synthesized from CP and maltodextrin.

^a–c^Means values in the same row are significantly different (*P* < 0.05).

Data are presented as mean ± standard deviation (n = 3).

#### 3.3.4 Electronic tongue of MRPs

To investigate the effect of the Maillard reaction on the taste attributes of CP, the taste characteristics of CP and MRPs were evaluated using an E-tongue analysis. Five taste sensors, including AHS, PKS, CTS, NMS, and ANS displayed specific responses to sourness, sweetness, saltiness, umami, and bitterness, respectively [48]. CP primarily exhibited sourness, followed by bitterness, saltiness, sweetness, and umami sensor intensities in a descending order. CPX and CPM showed reduced sourness compared with CP, while their bitterness and umami levels showed similar sensor intensities to CP (Fig. 4C). For saltiness and sweetness, CPX exhibited a decrease and increase, respectively, while CPM showed similar values to those of CP (Fig. 4C). It was assumed that the saccharides might independently affect the taste; particularly, the addition of the monosaccharide xylose imparted sweetness while masking saltiness. After the Maillard reaction, the sourness and bitterness scores of MCPX and MCPM were notably lower than those of CP, CPX, and CPM. Sourness was associated with the C-terminus of aspartic acid, which had an acidic side chain that could induce sourness [15]. Hydrophobic amino acids such as glycine, alanine, and methionine, along with bitter peptides, influence the bitterness of proteins. During the ultrasound-assisted Maillard reaction, these components might be decomposed by heat and pressure, resulting in decreased bitterness scores for MCPX and MCPM [15]. The umami sensor intensities of MCPX and MCPM were higher than those of CP, CPX, and CPM, with MCPX exhibiting the highest score at 7.9. The increase in umami after the Maillard reaction might be due to the release of umami free amino acids, such as glutamate, and low molecular weight peptides resulting from protein degradation [49]. Meanwhile, the saltiness sensor intensity of MCPX was lower than that of CP, while MCPM exhibited a similar saltiness score to CP. This trend was consistent with the results observed in CPX and CPM, respectively. Collectively, our E-tongue analysis of MRPs indicated that the Maillard reaction decreased the sourness and bitterness of CP while intensifying its umami taste.

### 3.4 Physicochemical and sensory characteristics of plant-based patties

In the previous section, the characteristics of MRPs were validated, focusing on their surface hydrophobicity, protein solubility, thermal stability, and taste attributes. As a result, to determine the compatibility of MCPX and MCPM as additives for PMAs, the physicochemical and sensory properties of PPs supplemented with CP, CPX, CPM, and MRPs (i.e., MCPX and MCPM) were evaluated.

#### 3.4.1 Color, rheological properties, and texture profile analysis of PPs

**Fig. 5:**
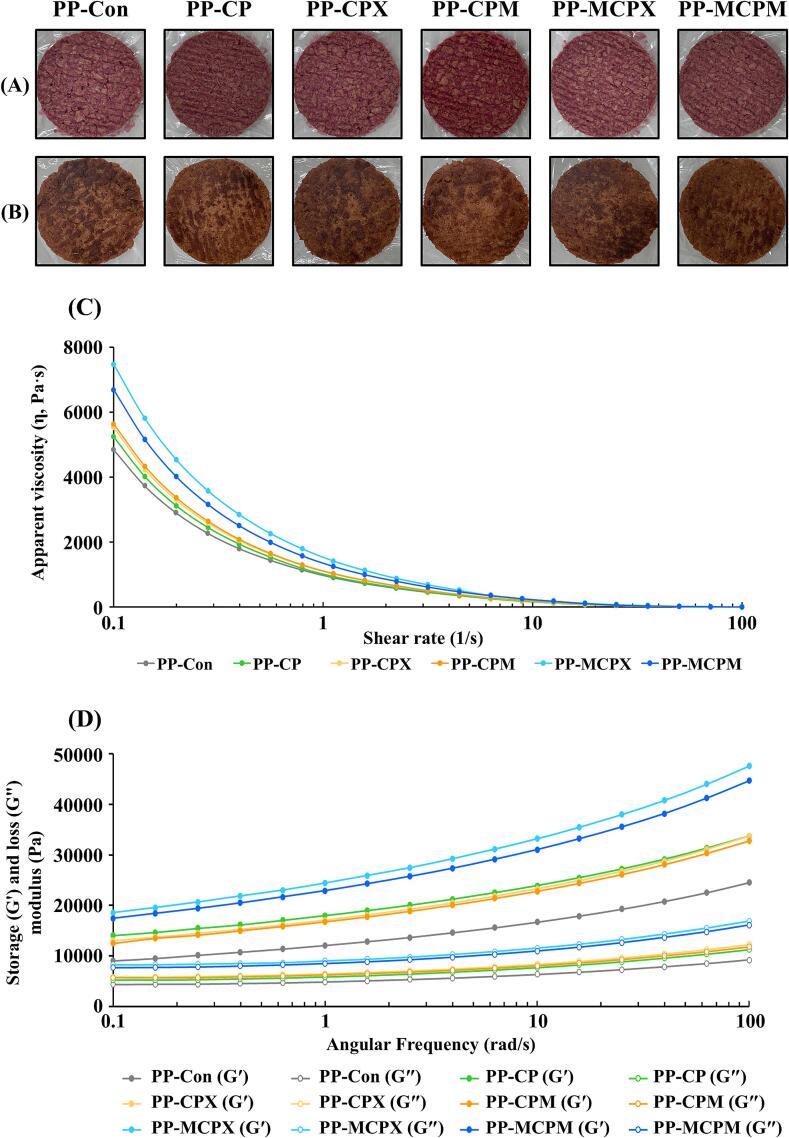
Physicochemical properties of plant-based patties (PPs) supplemented with *C. glutamicum*-derived protein (CP) and Maillard reaction products (MRPs). Visual appearance of (A) raw and (B) cooked PPs, (D) apparent viscosity at 25 °C, (C) viscoelasticity as a function of frequency in the range of 0.1–100 rad/s at 25 °C, and 1 % strain. Each colored line indicates a different sample. PP-Con: PP without CP and MRPs, PP-CP: PP supplemented with CP, PP-CPX: PP supplemented with CP and xylose mixture, PP-CPM: PP supplemented with CP and maltodextrin mixture, PP-MCPX: PP supplemented with MRPs synthesized from CP and xylose, PP-MCPM: PP supplemented with MRPs synthesized from CP and maltodextrin.

To investigate the physicochemical properties of MRPs on PPs, the color and rheological properties were evaluated (Fig. 5, Table S2). Meat color was a critical factor in assessing quality, significantly influencing consumer product preferences [50]. During the Maillard reaction, brown-colored melanoidins and acrylamide are produced. If present in excessive amounts, these compounds could lead to undesirable discoloration in meat products [51]. Our data showed that there were no significant differences in the color of PPs before and after cooking across all the PP groups (*P* > 0.05) (Fig. 5A and B, Table S2). These data indicate that the CP samples, particularly MCPX and MCPM, did not negatively affect the color of PPs before and after cooking.

To evaluate the rheological properties of PPs supplemented with CP, CPX, CPM, MCPX, and MCPM, their apparent viscosity and viscoelasticity were measured (Fig. 5C and D). Apparent viscosity refers to the change in viscosity with shear rate. All PPs exhibited shear-thinning behavior characteristic of non-Newtonian fluids, where the viscosity decreased with increasing shear rate (Fig. 5C). The addition of CP, CPX, and CPM slightly increased the apparent viscosity of PPs, likely due to the proteins and saccharides acting as thickeners. PP-MCPX and PP-MCPM showed a notable increase in the apparent viscosity compared to PP-Con, indicating that MCPX and MCPM had greater impacts on the viscosity of PPs, compared with the impacts of CP, CPX, and CPM. Similar to the results of apparent viscosity, non-destructive measurement of viscoelasticity revealed that PP-MCPX and PP-MCPM had higher G′ and G″ values than the other groups. These rheological properties were likely affected by various factors, such as the size and shape of droplets, as well as particle–particle interactions [39]. During the Maillard reaction, CP and saccharides formed large molecules through covalent bonding, leading to entanglement and interlocking within the PP, thereby increasing viscosity [39]. In MRPs, the high content of β-sheet structure, with their large surface area and weak hydration ability, facilitated mutual bonding between proteins and saccharides, enhancing gel strength [52]. Additionally, the unfolding of CP induced by the Maillard reaction exposed internal hydrophobic residues, decreasing delta H and promoting the binding of water and proteins, thereby increasing the strength of the PP gel [47]. Consequently, enhanced protein functions of MCPX and MCPM via the Maillard reaction contributed to improved rheological properties of the PPs.

Texture is a critical indicator for evaluating the quality of food products, alongside taste and flavor. To successfully mimic the sensory experience of meat in PPs, optimizing texture is essential. Table 3 showed the texture properties of PPs supplemented with MRPs. The hardness of PP-MCPX and PP-MCPM was significantly higher compared with the other groups (*P* < 0.05). MCPX and MCPM, characterized by a high β-sheet structure, facilitated interactions between proteins and saccharides while exposing internal hydrophobic residues. These interactions led to a reduction in delta H, thereby promoting water-protein-oil binding and strengthening the gel structure [47,53]. The increase in surface hydrophobicity enhanced the interaction between proteins and the interface layer, thereby increasing the compactness of the interface layer and potentially improving emulsification [54]. In addition, the increase in protein solubility promoted protein distribution at the oil–water interface, contributing to the formation of a thicker interface layer [54]. Our rheological property data demonstrated that PP-MCPX and PP-MCPM exhibited significantly higher gel strength compared with the other groups. (Fig. 5C and Fig. 5D). These results indicate that PP-MCPX and PP-MCPM effectively trap oil and water, resulting in the formation of a robust gel network within the patties. This high-strength gel structure was likely a critical factor contributing to the higher hardness values observed in the PPs. Previous studies have reported an increase in the hardness of Cantonese sausages attributed to the incorporation of MRPs derived from mechanically deboned chicken hydrolysate [55]. Meanwhile, PP-CPX and PP-CPM exhibited significantly higher hardness compared with PP-Con (*P* < 0.05). The cooking process, which involved surface heating at 150 °C and achieving an internal temperature of 80 °C, likely provided adequate conditions for the Maillard reaction to occur in CPX and CPM. However, MCPX and MCPM that had already undergone the Maillard reaction were believed to demonstrate superior binding capacities with proteins and water compared with CPX and CPM, thereby leading to higher hardness values. The springiness values of all groups showed no significant differences (*P* > 0.05). The addition of CPX, CPM, MCPX, and MCPM significantly enhanced the cohesiveness of PPs compared with PP-Con (*P* < 0.05), with PP-MCPX and PP-MCPM showing the highest values. Incorporating MCPX and MCPM into PPs facilitated the formation of a firm gel through interactions with proteins, water, and binders. This process contributed to the development of a stable internal network, resulting in increased cohesiveness. In addition to the hardness and cohesiveness, PP-MCPX and PP-MCPM showed significantly higher chewiness and gumminess values compared with other groups (*P* < 0.05). Collectively, the enhanced gel-forming capacity of MCPX and MCPM played a crucial role in significantly improving the textural properties of the PPs.

**Table 3:** Texture profile analysis of plant-based patties (PPs) supplemented with *C. glutamicum*-derived protein (CP) and Maillard reaction products (MRPs).

	PP-Con	PP-CP	PP-CPX	PP-CPM	PP-MCPX	PP-MCPM
Hardness (N)	8.23 ± 0.76^c^	8.87 ± 1.03^bc^	9.52 ± 0.53^b^	9.64 ± 0.32^b^	11.60 ± 1.13^a^	10.99 ± 1.22^a^
Springiness	0.74 ± 0.03	0.75 ± 0.03	0.76 ± 0.05	0.75 ± 0.04	0.75 ± 0.01	0.73 ± 0.00
Cohesiveness	0.44 ± 0.01^d^	0.46 ± 0.03^cd^	0.49 ± 0.04^abc^	0.48 ± 0.03^bc^	0.54 ± 0.04^a^	0.52 ± 0.02^ab^
Chewiness (N)	2.65 ± 0.24^c^	3.08 ± 0.48^bc^	3.51 ± 0.27^b^	3.46 ± 0.18^b^	4.70 ± 0.84^a^	4.18 ± 0.42^a^
Gumminess (N)	3.59 ± 0.34^c^	4.10 ± 0.51^bc^	4.65 ± 0.29^b^	4.64 ± 0.31^b^	6.24 ± 1.09^a^	5.74 ± 0.57^a^

PP-Con: PP without CP and MRPs, PP-CP: PP supplemented with CP, PP-CPX: PP supplemented with CP and xylose mixture, PP-CPM: PP supplemented with CP and maltodextrin mixture, PP-MCPX: PP supplemented with MRPs synthesized from CP and xylose, PP-MCPM: PP supplemented with MRPs synthesized from CP and maltodextrin.

^a–d^Means values in the same row are significantly different (*P* < 0.05).

Data are presented as mean ± standard deviation (n = 5).

#### 3.4.2 Sensory evaluation of PPs

**Fig. 6:**
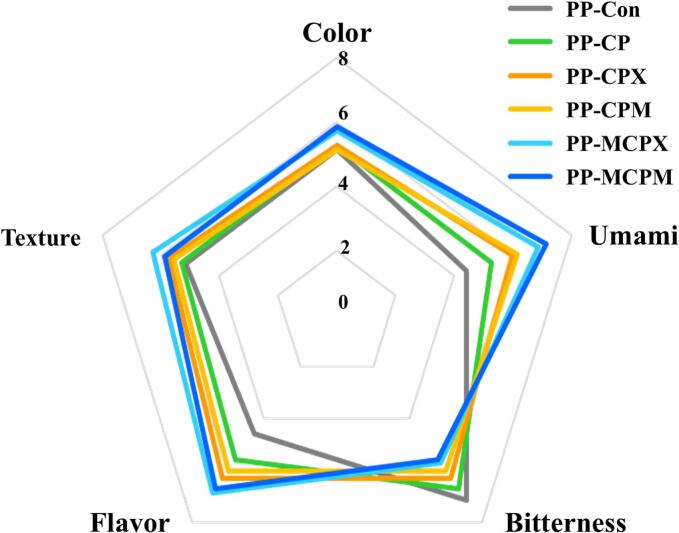
Sensory evaluation of plant-based patties (PPs) supplemented with *C. glutamicum*-derived protein (CP) and Maillard reaction products (MRPs). Each colored line indicates a different sample. PP-Con: PP without CP and MRPs, PP-CP: PP supplemented with CP, PP-CPX: PP supplemented with CP and xylose mixture, PP-CPM: PP supplemented with CP and maltodextrin mixture, PP-MCPX: PP supplemented with MRPs synthesized from CP and xylose, PP-MCPM: PP supplemented with MRPs synthesized from CP and maltodextrin. The experiment was conducted with seven panelists (n = 7).

Successful production of PMAs relis on mitigating the inherent bitterness of plant proteins and amplifying umami flavor to mimic the taste of real meat [26,56]. Our E-tongue analysis of MRPs revealed that MCPX and MCPM exhibited high umami values and low bitterness values (Fig. 4C). Therefore, a sensory evaluation of PPs supplemented with MRPs was performed to assess attributes including color, umami, bitterness, flavor, and texture (Fig. 6). The color scores of PP-MCPX and PP-MCPM were higher than those of the other groups; however, the differences were not significant (*P* > 0.05). The color data of PPs also revealed that the L*, a*, and b* values did not exhibit significant differences among the groups (*P* > 0.05, Table S2). Therefore, our findings suggested that the incorporation of MCPX and MCPM did not adversely affect the color characteristics of the PPs. PP-MCPX and PP-MCPM exhibited significantly higher umami and flavor scores and lower bitterness compared with PP-Con (*P* < 0.05). Our E-tongue analysis also demonstrated that MRPs have higher umami and saltiness levels and lower bitterness compared with the other groups (Fig. 4C). A recent study suggested that Maillard reactions involving aspartic acid and hydrophobic amino acids may contribute to reduced bitterness [15]. The increased umami content in these formulations might improve palatability and reduce bitterness [57]. PP-MCPX and PP-MCPM demonstrated significantly higher texture scores compared with PP-Con (*P* < 0.05). This enhancement was attributed to the superior gel-forming ability of MCPX and MCPM, leading to improved textural properties in the PPs. Interestingly, a trend towards increased umami and flavor scores, coupled with a slight decrease in bitterness, was observed in PP-CPX, and PP-CPM compared with PP-CP and PP-Con. These data suggested that PP-CPX and PP-CPM underwent Maillard reactions during cooking, enhancing their taste compared with PP-CP. However, the taste enhancement observed in PP-CPX and PP-CPM was less pronounced than that of PP-MCPX and PP-MCPM. Collectively, the sensory evaluation data provided strong evidence that the incorporation of MCPX and MCPM significantly enhanced the sensory quality of PPs. This enhancement was primarily attributed to a reduction in negative taste attributes, coupled with a notable improvement in umami perception and textural characteristics.

## 4 Conclusions

This study aimed to enhance the function and taste attributes of CP through the ultrasound-assisted Maillard reaction, enabling its application in PPs. The optimal conditions for the Maillard reaction were determined to be a CP–saccharide mass ratio of 4:1, ultrasound power of 400 W, ultrasound time of 45 min, and ultrasound temperature of 70 °C. Under these selected conditions, MRPs (i.e., MCPX and MCPM) synthesized from CP and saccharides exhibited significant enhancements in GD, browning intensity, and structural stability (*P* < 0.05). The enhanced surface hydrophobicity, protein solubility, and thermal stability observed in MCPX and MCPM resulted from the formation of hydrogen bonds, hydrophobic interactions, and β-sheet structures. The taste attributes of MRPs contributed to a reduction in sourness and bitterness while enhancing umami, making them more suitable for food applications. In addition, incorporating MRPs into PPs significantly improved their texture properties and enhanced taste attributes without adversely affecting color. In conclusion, our study demonstrated that the physical and sensory properties of CP were significantly enhanced through the Maillard reaction. Due to its high lysine and glutamic acid content, CP was particularly conductive to Maillard reaction and showed potential as a flavor-enhancing food additive. As the first study to investigate the application of MRPs in PMA, our findings establish ultrasound-assisted Maillard reaction as a highly effective approach for optimizing CP in the formulation of high-quality products. Further investigations should focus on the development of CP hydrolysate-derived MRPs, with particular consideration given to the potential formation of advanced glycation end products and their implications for product safety.

## CRediT authorship contribution statement

**Jong Hyeon Han:** Writing – review & editing, Writing – original draft, Methodology, Investigation, Formal analysis, Conceptualization. **Dong Hyun Keum:** Writing – review & editing, Methodology. **Hyun Ju Lee:** Writing – review & editing, Methodology. **Yea-Ji Kim:** Writing – review & editing, Methodology, Investigation. **Hyun Su Jung:** Writing – review & editing, Methodology. **Do Hyun Kim:** Writing – review & editing, Methodology. **Hyuk Cheol Kwon:** Writing – review & editing, Methodology, Investigation. **Dong-Min Shin:** Writing – review & editing, Methodology. **Sung Gu Han:** Writing – review & editing, Writing – original draft, Conceptualization.

## Declaration of competing interest

The authors declare that they have no known competing financial interests or personal relationships that could have appeared to influence the work reported in this paper.
