# Supplementary material for: Ultrasound-assisted Maillard reaction of Corynebacterium glutamicum protein: Impact on structure, taste, and plant-based meat applications
Source: Ultrason Sonochem. 2025 Jun 7;120:107424. doi: 10.1016/j.ultsonch.2025.107424 (PMC12182386; doi:10.1016/j.ultsonch.2025.107424)
Supplement: Supplementary Data 1 [file mmc1.docx]

**Supplementary materials**


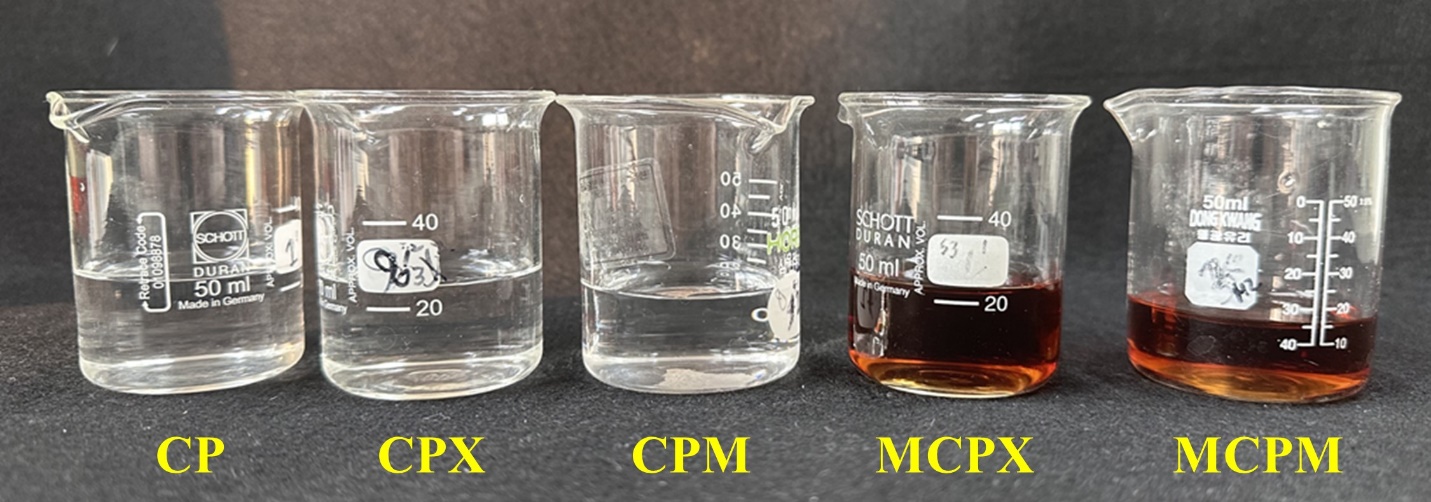


**Fig. S1.** Visual appearance of *C. glutamicum*-derived protein and Maillard reaction products (MRPs). CP: *C. glutamicum*-derived protein, CPX: CP and xylose mixture, CPM: CP and maltodextrin mixture, MCPX: MRPs synthesized from CP and xylose, MCPM: MRPs synthesized from CP and maltodextrin.

**Table S1** Formulation of plant-based patties (PPs) supplemented with *C. glutamicum*-derived protein (CP) and Maillard reaction products (MRPs) synthesized from CP and saccharides

| Ingredients (%) | Plant-based patty groups^1)^ | | | | | |
| --- | --- | --- | --- | --- | --- | --- |
|  | PP-Con | PP-CP | PP-CPX | PP-CPM | PP-MCPX | PP-MCPM |
| Textured pea protein | 20 | 20 | 20 | 20 | 20 | 20 |
| Water | 56.65 | 55.65 | 55.65 | 55.65 | 55.65 | 55.65 |
| Methylcellulose | 2 | 2 | 2 | 2 | 2 | 2 |
| κ-Carrageenan | 2 | 2 | 2 | 2 | 2 | 2 |
| Potato starch | 1.5 | 1.5 | 1.5 | 1.5 | 1.5 | 1.5 |
| Salt | 1.1 | 1.1 | 1.1 | 1.1 | 1.1 | 1.1 |
| Beet powder | 0.75 | 0.75 | 0.75 | 0.75 | 0.75 | 0.75 |
| Isolated pea protein | 4 | 4 | 4 | 4 | 4 | 4 |
| Canola oil | 6 | 6 | 6 | 6 | 6 | 6 |
| Coconut oil | 6 | 6 | 6 | 6 | 6 | 6 |
| CP | 0 | 1 | 0 | 0 | 0 | 0 |
| CPX | 0 | 0 | 1 | 0 | 0 | 0 |
| CPM | 0 | 0 | 0 | 1 | 0 | 0 |
| MCPX | 0 | 0 | 0 | 0 | 1 | 0 |
| MCPM | 0 | 0 | 0 | 0 | 0 | 1 |
| Total | 100 | 100 | 100 | 100 | 100 | 100 |

^1)^ PP-Con: PP without CP and MRPs, PP-CP: PP supplemented with CP, PP-CPX: PP supplemented with CP and xylose mixture, PP-CPM: PP supplemented with CP and maltodextrin mixture, PP-MCPX: PP supplemented with MRPs synthesized from CP and xylose, PP-MCPM: PP supplemented with MRPs synthesized from CP and maltodextrin.

**Table S2** Color of plant-based patties (PPs) supplemented with *C. glutamicum*-derived protein (CP) and Maillard reaction products (MRPs).

| Parameters | | PP-Con | PP-CP | PP-CPX | PP-CPM | PP-MCPX | PP-MCPM |
| --- | --- | --- | --- | --- | --- | --- | --- |
| Raw | *L*^*^ | 50.67±0.95 | 50.47±0.96 | 50.11±1.24 | 50.79±1.33 | 51.03±0.81 | 51.18±0.38 |
|  | *a*^*^ | 22.23±0.46 | 22.23±0.46 | 22.12±0.30 | 22.23±0.46 | 22.24±0.54 | 22.26±0.75 |
|  | *b*^*^ | 12.55±0.62 | 12.65±0.39 | 12.47±0.89 | 12.59±0.58 | 12.89±0.54 | 12.79±0.60 |
| Cooked | *L*^*^ | 42.97±1.91 | 43.48±1.59 | 42.06±1.93 | 42.88±1.18 | 42.13±1.92 | 43.30±1.71 |
|  | *a*^*^ | 10.79±0.39 | 10.87±0.39 | 10.63±0.84 | 10.99±0.49 | 10.86±0.92 | 10.78±0.83 |
|  | *b*^*^ | 15.37±1.29 | 15.67±2.32 | 15.02±1.49 | 16.06±1.12 | 15.01±1.87 | 15.28±2.13 |

PP-Con: PP without CP and MRPs, PP-CP: PP supplemented with CP, PP-CPX: PP supplemented with CP and xylose mixture, PP-CPM: PP supplemented with CP and maltodextrin mixture, PP-MCPX: PP supplemented with MRPs synthesized from CP and xylose, PP-MCPM: PP supplemented with MRPs synthesized from CP and maltodextrin.

Data are presented as mean ± standard deviation (n = 6).
